# Sporotrichosis Cases in Commercial Insurance Data, United States, 2012–2018

**DOI:** 10.3201/eid2611.201693

**Published:** 2020-11

**Authors:** Kaitlin Benedict, Brendan R. Jackson

**Affiliations:** Centers for Disease Control and Prevention, Atlanta, Georgia, USA

**Keywords:** sporotrichosis, geography, health insurance, United States, fungi, zoonoses, sphagnum moss

## Abstract

The geographic distribution of sporotrichosis in the United States is largely unknown. In a large commercial health insurance database, sporotrichosis was rare but most frequently occurred in southern and south-central states. Knowledge about where sporotrichosis is most likely to occur is essential for increasing clinician awareness of this rare fungal disease.

Sporotrichosis is an infection caused by the fungus *Sporothrix*. The infection typically follows cutaneous inoculation and involves the skin, subcutaneous tissue, and lymph nodes; pulmonary or disseminated disease occurs less frequently and usually affects immunocompromised persons (*1*). *Sporothrix* exists nearly worldwide in soil and decaying plant matter, but many unanswered questions remain about its precise ecologic niche (*2*). In the United States, its geographic distribution is poorly understood. Knowledge about where sporotrichosis is most likely to occur can help healthcare providers recognize and treat it earlier and help public health officials focus prevention messages.

We used the MarketScan Research Databases (IBM, https://www.ibm.com) to examine the geographic distribution of sporotrichosis in the United States. These databases comprise health insurance claims data from outpatient visits, prescriptions, and hospitalizations for employees, dependents, and retirees throughout the United States. In 2018, the databases contained records for »27 million persons. MarketScan data are fully de-identified; thus, the Centers for Disease Control and Prevention institutional review board did not need to approve this study.

To query the database, we used Treatment Pathways (IBM), a web-based platform, that comprises data from persons with health insurance plans that contributed prescription drug information to the MarketScan databases. We used data from February 1, 2012–December 31, 2018, to identify sporotrichosis patients using code 117.1 from the International Classification of Diseases (ICD), Ninth Revision, Clinical Modification and code B42 from the ICD, 10th Revision, Clinical Modification (ICD-10-CM). We used the primary beneficiary’s state of residence to calculate average annual state-specific rates per 1 million MarketScan enrollees. We evaluated underlying conditions on or in the month before sporotrichosis diagnosis, demographic features, and type of sporotrichosis. 

Of ≈76 million unique patients during 2012–2018, 1,322 had a sporotrichosis diagnosis code. For 1,236 (93.5%) of those, information was available about state of residence. The average annual rate of sporotrichosis cases per 1 million enrollees was highest in Oklahoma (6.1), Michigan (3.9), Kansas (3.5), and Kentucky (3.5) (Figure). Nationwide, the average annual rate was 2.0 cases/1 million enrollees.

**Figure:**
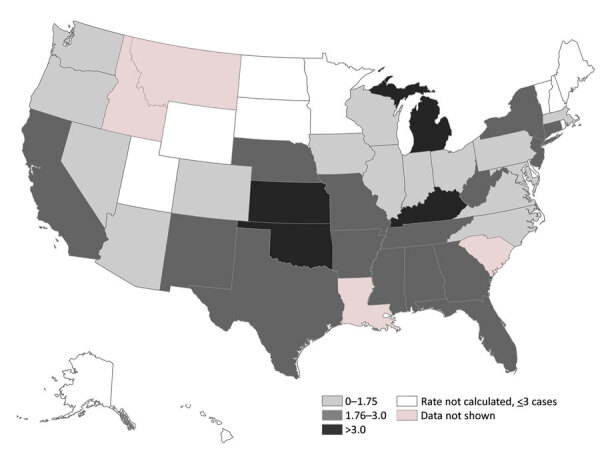
Average annual sporotrichosis rate per 1 million MarketScan enrollees, United States, 2012–2018. MarketScan (IBM, https://www.ibm.com) data are a subset of privately insured persons in the United States and are not necessarily representative of persons with other types of health insurance or of persons without insurance. To avoid potentially unreliable estimates, the rates for states with <3 cases were not calculated. MarketScan does not permit state-level analyses for certain states.

For the 1,252 patients continuously enrolled during the month before their diagnosis, median age was 54 years; most (62%) patients were female (Table). The most common underlying conditions we evaluated were diabetes (7%), immune-mediated inflammatory disease (3%), and chronic obstructive pulmonary disease (2%). Among 514 patients with sporotrichosis ICD-10-CM codes, specific types included lymphocutaneous in 13% and unspecified forms in 69%. Thirty-seven (3%) patients were hospitalized at diagnosis.

**Table:** Characteristics of 1,252 patients with sporotrichosis, United States, 2012–2018*

Characteristic	No. (%)
Age group, y†	
0–17	46 (4)
18–34	153 (12)
35–44	154 (12)
45–54	305 (24)
55–64	402 (32)
>65	192 (15)
Sex	
F	774 (62)
M	478 (38)
Residence in a rural area	184 (15)
Type of sporotrichosis	
Lymphocutaneous‡	68 (13)
Pulmonary§	27 (5)
Other forms¶	71 (14)
Unspecified#	357 (69)
Underlying conditions on or in the month before sporotrichosis diagnosis	
Alcohol related disorders**	9 (0.7)
Immune-mediated inflammatory disease††	39 (3)
Chronic obstructive pulmonary disease‡‡	26 (2)
Diabetes§§	90 (7)
HIV disease¶	5 (0.4)
Solid organ or stem cell transplant##	5 (0.4)
Hematologic malignancy***	7 (0.6)
*Diagnosis codes for sporotrichosis are as follows: ICD-9-CM code 117.1 and ICD-10-CM B42. ICD-9-CM, International Classification of Diseases, Ninth Revision, Clinical Modification; ICD-10-CM, International Classification of Diseases, 10th Revision, Clinical Modification. †Median age 54 y (range 0–93 y). ‡ICD-10-CM code B42.1. §ICD-10-CM code B42.0. ¶ICD-10-CM codes B42.7, B42.81, B42.82, B42.89. #ICD-10-CM code B42.9. **ICD-9-CM codes 291, 303, 305.0, 571.0–571.3; ICD-10-CM codes F10, K70. ††ICD-9-CM codes 555, 556, 696.0, 696.1, 696.8, 714.0, 714.2; ICD-10-CM codes K50, K51, L40, M023, M05, M06, M08, M33, M352, M45. ‡‡ICD-9-CM codes 490–492, 494, 496; ICD-10-CM codes J41–J44. §§ICD-9-CM codes 249–250; ICD-10 codes E08–E11. ¶¶ICD-9-CM code 042; ICD-10-CM code B20. ##ICD-9-CM codes V42 (excluding V42.3–V42.5), 996.8; ICD-10-CM codes T86, Z94 (excluding Z94.5– Z94.7). ***ICD-9-CM codes 200–208; ICD-10-CM codes C81–C86, C88, C90–C96.	

Although sporotrichosis occurred rarely in this large sample of privately insured patients, it was most common in the southern and south-central United States. The US geographic distribution of sporotrichosis has not been well described since the 1940s, when most cases were observed in the Mississippi River basin, with the highest frequencies in North Dakota, Nebraska, Wisconsin, Kansas, and Missouri (*3*). Reasons for the low rates we found in North Dakota and Wisconsin and the high rates in Michigan are unknown. Historically, Wisconsin-grown sphagnum moss has been the most common source of sporotrichosis outbreaks. At least 8 published outbreaks implicated Wisconsin moss shipped to other states for use in topiaries or packing material for tree seedlings (*4*,*5*). Since the late 1990s, sporotrichosis outbreaks appeared absent from published literature (*4*). Industry changes in harvesting sources or processing methods might play a role in the absence of outbreaks (*5*). Public health officials might not detect or investigate these outbreaks, as no routine surveillance for sporotrichosis exists. Our observation of higher sporotrichosis rates in Oklahoma and Kansas is consistent with past outbreaks linked to contaminated hay in those states (*6*–*8*).

In our analysis, sporotrichosis more frequently affected older women. These results possibly reflected differences in care-seeking behavior or exposures. Underlying conditions were uncommon, suggesting that most cases occurred in previously healthy persons. This finding was consistent with lymphocutaneous disease resulting from traumatic inoculation.

Our findings are subject to several limitations. The primary limitation was that patients’ states of residence might not represent the exposure location or the original environmental source of *Sporothrix*. Undetected cases and potential case misclassification are inherent limitations of administrative data. Diagnosis codes might substantially underestimate the true number of cases because affected persons might not seek care for self-limiting sporotrichosis. In addition, sporotrichosis might not have been correctly diagnosed or coded. Furthermore, administrative data often lack detail (i.e., differentiating between different sporotrichosis forms is not possible in ICD’s Ninth Revision, Clinical Modification, and was not frequently used in ICD-10-CM). However, MarketScan is one of the few data sources large enough to provide a sufficient number of sporotrichosis cases to analyze at a subregional level.

Knowledge about where sporotrichosis is most likely to occur is essential for increasing clinician awareness of this rare disease. Increased awareness might lead to faster diagnosis and treatment with antifungal medication, which most sporotrichosis patients need (*1*). Understanding the distribution of sporotrichosis is also essential for understanding emerging sources of infection. Parts of Latin America are experiencing a large and growing epidemic of severe sporotrichosis caused by cat-transmitted *S. brasiliensis* (*9*). This emerging infection provides further evidence of the need for ongoing monitoring.

## References

[1] Kauffman CA, Bustamante B, Chapman SW, Pappas PG; Infectious Diseases Society of America. Clinical practice guidelines for the management of sporotrichosis: 2007 update by the Infectious Diseases Society of America. Clin Infect Dis. 2007;45:1255–65. 10.1086/522765 17968818

[2] Chakrabarti A, Bonifaz A, Gutierrez-Galhardo MC, Mochizuki T, Li S. Global epidemiology of sporotrichosis. Med Mycol. 2015;53:3–14. 10.1093/mmy/myu062 25526781

[3] Gastineau F, Spolyar L, Haynes E. Sporotrichosis: report of six cases among florists. J Am Med Assoc. 1941;117:1074–7. 10.1001/jama.1941.02820390016005

[4] Hajjeh R, McDonnell S, Reef S, Licitra C, Hankins M, Toth B, et al. Outbreak of sporotrichosis among tree nursery workers. J Infect Dis. 1997;176:499–504. 10.1086/514070 9237718

[5] Coles FB, Schuchat A, Hibbs JR, Kondracki SF, Salkin IF, Dixon DM, et al. A multistate outbreak of sporotrichosis associated with sphagnum moss. Am J Epidemiol. 1992;136:475–87. 10.1093/oxfordjournals.aje.a116521 1415167

[6] Dooley DP, Bostic PS, Beckius ML. Spook house sporotrichosis. A point-source outbreak of sporotrichosis associated with hay bale props in a Halloween haunted-house. Arch Intern Med. 1997;157:1885–7. 10.1001/archinte.1997.00440370135014 9290549

[7] Centers for Disease Control (CDC). Sporotrichosis among hay-mulching workers—Oklahoma, New Mexico. MMWR Morb Mortal Wkly Rep. 1984;33:682–3. 6438470

[8] Dahl BA, Silberfarb PM, Sarosi GA, Weeks RJ, Tosh FE. Sporotrichosis in children. Report of an epidemic. JAMA. 1971;215:1980–2. 10.1001/jama.1971.03180250072022 5107842

[9] Gremião IDF, Oliveira MME, Monteiro de Miranda LH, Saraiva Freitas DF, Pereira SA. Geographic expansion of sporotrichosis, Brazil. Emerg Infect Dis. 2020;26:621–4. 10.3201/eid2603.190803 32091376 PMC7045854

